# The After-Effects of Infantile Palsy

**Published:** 1909-09-18

**Authors:** 


### Diseases of Children.

### THE AFTER-EFFECTS OF INFANTILE PALSY.

About three-quarters of the cases of infantile paralysis occur during the four months from June to September, that is, in the hot months of the year. The time is, therefore, appropriate to draw attention to the sequelæ of the disease and the means whereby its effects can be minimised or prevented. It is in young children we must be especially on the look-out for its occurrence. Ninety per cent. of the cases are under five years of age at the onset and most of them are infants, that is, under two-and-a-half years of age. It is most common at the age of twelve to eighteen months. Frequently it breaks out in epidemics or in several children of the same family.

The most common mode of onset is a febrile attack, with gastro-intestinal symptoms, for some hours or days before the onset of the palsy. Sometimes there is no previous sign of ill-health and the paralysis is the first thing noted, perhaps on waking up in the morning. On account of its mode of onset it is frequently mistaken for simple gastric disturbance or the incidence of a specific fever. This does not bring any credit to a medical practitioner who has not suspected the possibility of making an erroneous diagnosis, more especially if he has ascribed the paralysis of the limb or limbs to the weakness and inertia of illness, and soothed the mother with the idea that the whole trouble is due to teething.

The initial loss of power is greater than the permanent palsy, and improvement sets in a few days after the acute symptoms and the fever have subsided. This is known as the stage of quiescence, fixation, or regression. Distinct wasting begins in two or three weeks and steadily progresses. In partial recovery of the muscles some of the fibres remain paralysed, atrophy, and are replaced by fibrous and adipose tissue. The tone of the muscle is diminished and it becomes stretched by the antagonistic action of its opponents. This is still more marked if the muscle is completely paralysed. Subsequently the opponent muscles are physically shrunken by over-contraction and produce deformity which can easily be overcome in the early stages. If it is neglected, it ends in pathological shrinking, fibrosis, contractures, and permanent deformities. Eventually fascia, ligaments, joints, and bones become involved.

It is to direct attention to the possibility of preventing the worst of these deformities and limiting the incidence of the milder types that this article is written. As a simple illustration we may take paralysis of one lower limb. To a certain extent the deformity depends on extraneous causes. Thus, the weight of the bed-clothes is a serious factor in the production of talipes equinus. It is, therefore, of the utmost importance that the use of a cradle is insisted on. This alone is insufficient. It is necessary to apply a padded tin splint to the foot and leg, so that the foot is kept at a right angle to the leg. This prevents the stretching of the paralysed muscles, a stretching which is such a serious detriment to successful treatment, for stretched muscles recover badly. It is almost equally important to prevent the stretching of tendons and ligaments. If not, the characteristic "flail-joint" is produced, and the surgeon is reduced to arthrodesis as the only method of obtaining a useful limb, or the patient has to wear a mechanical apparatus for the rest of his life. Although these measures help to prevent deformity, they do not cure or directly benefit the paralysis. For this we must rely on steady persistence in the use of massage, electricity, and exercises. Mechanical splints and apparatus help to prevent deformity and to overcome that already present, but if they are worn continuously the muscles continue to atrophy from disuse, or at any rate do not improve.

Electrical treatment must be continued for six months, using a galvanic current of such a strength as barely to induce contraction; or the galvanic bath if both the lower limbs are affected. Lewis Jones advocates the sinusoidal current from the main in electric baths. A weak faradic current is sometimes employed for those muscles which react to it, but some observers are inclined to think it increases the tendency to atrophy. Electrical treatment may be continued for a year, if there is any improvement. At first it is applied on alternate days, then daily for some months, finally two or three times a week. During all this time and for a total period of two years friction and massage must be systematically carried on, in order to maintain muscular nutrition and prevent deformity. Begin passive and active movements as soon as the palsy is quiescent. No doubt a skilled masseur is an advantage, but, if unavailable, almost as good results can be obtained by training the mother or nurse in the method.

After the lapse of two years from the onset we must rely on the effects of growth and systematic education of the muscles. Here again it is not essential to transfer the patient to the tender mercies, and sometimes expensive ones, of a specialist or a foreign professor of gymnastics. If the doctor will take the trouble to ascertain which muscles are defective, he can soon devise and teach the exercises suitable for the different cases, and the nurse or mother can see that they are carried out. Particular care must be taken in paralysis of the spinal muscles that the proper ones are exercised.

After the lapse of six months, and rarely before, surgical measures may be needed to correct deformity and enable the child to walk better. It is important to get the foot flat on the ground. Tenotomy, with or without shortening of the tendon, is of temporary benefit. Many cases relapse. Better results are obtained by transplantation of tendon or muscle, and by muscle grafting. Everything should be done to encourage the child to make use of the affected limb, for it is quite astonishing what good results can be obtained by preventive measures and the steady persistence in treatment on the lines here recommended.

